# IL-6 serum level, ARDS, and AKI as risk factors for the COVID-19 infection’s mortality in children

**DOI:** 10.1371/journal.pone.0293639

**Published:** 2023-10-27

**Authors:** Idham Jaya Ganda, Try Kartika Eka Putri, Syarifuddin Rauf, Amiruddin Laompo, Ninny Meutia Pelupessy, Sitti Aizah Lawang, Nadirah Rasyid Ridha, Bahrul Fikri, Muhammad Nasrum Massi

**Affiliations:** 1 Emergency and Pediatric Intensive Care Division, Department of Pediatrics, Faculty of Medicine Hasanuddin University, Makassar, Indonesia; 2 Child Health Department, DR Wahiddin Sudirohusodo Hospital, Makassar, Indonesia; 3 Department of Pediatrics, Faculty of Medicine Hasanuddin University, Makassar, Indonesia; 4 Nephrology Division, Department of Pediatrics, Faculty of Medicine Hasanuddin University, Makassar, Indonesia; 5 Respirology Division, Department of Pediatrics, Faculty of Medicine Hasanuddin University, Makassar, Indonesia; 6 Infection & Tropical Disease Division, Department of Pediatrics, Faculty of Medicine Hasanuddin University, Makassar, Indonesia; 7 Hematology-oncology Division, Department of Pediatrics, Faculty of Medicine Hasanuddin University, Makassar, Indonesia; 8 Allergy & Immunology Division, Department of Pediatrics, Faculty of Medicine Hasanuddin University, Makassar, Indonesia; 9 Department of Microbiology, Faculty of Medicine Hasanuddin University, Makassar, Indonesia; Guilan University of Medical Sciences, ISLAMIC REPUBLIC OF IRAN

## Abstract

**Introduction:**

Dysregulated immune responses are developed in Coronavirus disease-2019 (COVID-19) and Interleukin-6 (IL-6) levels are reflecting the severity of the clinical presentation. This study aimed to analyze IL-6 serum level, Acute Respiratory Distress Syndrome (ARDS), and Acute Kidney Injury (AKI) as risk factors for mortality in children with COVID-19.

**Methods:**

This prospective cohort study was conducted on children with COVID-19 infection confirmed by Real Time Polymerase Chain Reaction (RT-PCR) who were admitted to infection center at Dr. Wahidin Sudirohusodo Hospital from September 2021 to September 2022. Subjects were selected using the consecutive sampling method.

**Results:**

A total of 2,060 COVID-19 RT-PCR tests were performed, and 1,065 children were confirmed positive. There were 291 cases that met the inclusion criteria, with 28.52 percent non-survives and 71.48% survives. The risk factors for mortality were IL-6, ARDS, AKI, Prothrombin Time / Activated Partial Thromboplastin Time (PT/aPTT), oxygen saturation, Absolut lymphocyte count (ALC), leukocytes, Length of Stay (LOS), and nutritional status (p<0.05). IL-6 levels increased in all patients (23.48–252.58 pq/ml). COVID-19 patients with AKI, ARDS, low oxygen saturation and thrombocytopenia had the highest levels of IL-6 (p 0.05). The IL-6 cut-off point was >80.97 pg/ml with 93% sensitivity and 90% specificity. Area Under Curve was 0.981 (95% CI), 0.960–1.000). A multivariate analysis showed IL-6 levels with OR 18.570 (95% CI 5.320–64.803), ARDS with Odds Ratio (OR) 10.177, (95% Confidence Interval (CI) 1.310–9.040), and AKI with OR 3.220 (95% CI 1.070–10.362). A combination of increased IL-6, ARDS, and AKI can predict a mortality probability as high as 98.3%.

**Conclusion:**

IL-6, ARDS, and AKI are risk factors for mortality in children with COVID-19. IL-6 level was the highest mortality risk factor.

## Introduction

Coronavirus disease-2019 (COVID-19) infection is caused by SARS-CoV-2, which binds Angiotensin Converting Enzyme (ACE-2) receptors in all parts of the human body, especially in respiratory epithelial cells. Most cases have mild symptoms, about 14% develop into severe cases requiring hospitalization, and 5% are treated in intensive care [1]. The Indonesian Pediatrician Society reported 37,706 confirmed cases of COVID-19 in Indonesian children from March to December 2020, with the highest mortality rate at the age of 10–18 years (26%), then 1–5 years (23%), 29 days to <12 months (23%), 0–28 days (15%), and ages 6- <10 years (13%) [2]. The main challenges in the COVID-19 management were the absence of an appropriate antiviral drug to treat the SARS-CoV-2 virus and the wide-ranging spectrum of clinical manifestations from asymptomatic to severe and critical symptoms. In addition, the burdens of COVID-19 is high costs and mortality rates [3].

Interleukin-6 (IL-6) is a very important immunological molecule because of its pleiotropic effect. IL-6 is produced rapidly and transiently in response to infection and tissue injury [4]. Increased expressions of IL-6 in COVID-19 infection causes damage to lung tissue and development of infection [5]. Several studies showed that IL-6 is better at reflecting the severity of the clinical presentation of COVID-19 and is relatively more consistent than other inflammatory markers. An increase in cytokines such as IL-6 will trigger a cytokine storm [6]. Cytokine storm is known to be responsible for severe clinical manifestation of COVID-19 which eventually led to death [7]. COVID-19 infection is characterized by a massive proinflammatory cytokine response or cytokine storm that causes Acute Respiratory Distress Syndrome (ARDS) through a process of damage to the alveolar epithelium and microvascular endothelium resulting in an increase in the permeability of the alveolar and capillary barriers. This condition causes extravasation of fluid into the alveolar space. In addition, damage to the alveolar epithelium (pneumocytes type 1 and type 2) will cause disruption of intracellular metabolic processes, ion transport, disruption of surfactant production and trigger the formation of fibrosis which will cause disruption of oxygen diffusion and perfusion resulting in hypoxemia in patients [8].

COVID-19 can initiate Acute Kidney Injury (AKI) through vascular disturbances due to induction of coagulopathy, invasion of the COVID-19 virus into kidney tubular cells and podocytes, increased levels of cytokines, and other non-specific factors of kidney injury (pulmonary/cardiac dysfunction, increased Positive End Expiratory Pressure (PEEP), nephrotoxic drugs, fluid restriction, and hemodynamic instability) [9]. It is important to conduct research to analyze these three parameters to establish the role of increased IL-6, AKI, and ARDS on COVID-19 mortality in children. Therefore, this study aimed to analyze Those parameters as risk factors for mortality of COVID-19 in children. There are only a few studies on mortality risk factors of COVID-19 in children, and none have been performed in Eastern Indonesia.

## Materials and methods

### Study design

This is an observational prospective cohort study conducted at the referral center of Dr. Wahidin Sudirohusodo Hospital Makassar, South Sulawesi, Eastern Indonesia, from September 2021 to September 2022. Inclusion criteria were children aging 1 month to <18-year-old, confirmed to have COVID-19 infection, first onset of COVID-19, have not administered with COVID-19 vaccine, and have not treated in Isolation Ward of Infection Center in Dr Wahidin Sudirohusodo Hospital. Exclusion criteria were the patient had anti-IL-6 therapy before recruitment, nephrotic syndrome, chronic kidney failure, malignancy, sepsis, hypovolemic and cardiogenic shock, and burn injury. The determination of estimated sample size was performed using the sample size formula for proportion comparison, if RR 1.75 is considered significant, the proportion in the COVID-19 with AKI is 20%, with a significant value of 0.05 and a power of 80%, the sample size is calculated as follow: (1.96√2(0.4X0.6) + 0.842√0.5X0.5 + 0.3X0.7)2/(0.5–0.3)2 = 40, so the minimum number of samples is 40 samples. Sample selection by consecutive sampling.

Diagnosis of COVID-19 was established with RT-PCR by taking viral sample from nasopharyngeal and oropharyngeal swab. Patients were confirmed as positive COVID-19 if RNA-SARS-COV-2 detected. Subjects were observed from admission until outcome were achieved (survive and non-survive). History taking, physical examination, oxygen saturation, radiological examination, laboratory examination (routine blood test, type count, blood gas analysis, urea, creatinine, electrolytes, PT / aPTT, and urine production were taken. All other primary diseases were recorded. Level of IL-6 was checked in first day of hospitalization by taking 3 ml of vein blood. The samples were centrifuged for 30 minutes and were put in a cooler box at a temperature of 2–8 °C to be taken to a negative pressure laboratory. IL-6 levels were measured by enzyme linked immunosorbent assay (ELISA), research diagnostic, BioSource International, In, immunoassay kit, California). A kit was prepared for the examination of IL-6 levels and the sample is heated naturally at room temperature for 30 minutes. Sample was placed on the plate then given reagents and ELISA liquid then incubated for 60 minutes with temperature 37 °C. The plate was washed 5 times before being incubated for 10 minutes with temperature 37 °C until the color changed. After drying for 10 minutes, the sample was ready to be analyzed. The IL-6 levels were measured in picograms per milliliter.

Glasgow coma scale was assessed on the first day of hospitalization. Malnutrition in children aged under 5 years was defined as follows; moderately wasted was weight-for-height (W/H) <-2 standard deviations (SD) of the WHO Child Growth Standards median, severely wasted was W/H <-3 SD, overweight was W/H > +2 SD, and obesity was W/H >+3 SD. Malnutrion in children aged 5 years and older was defined as follows; underweight was below the 5th percentile of the CDC Body mass index (BMI) to age, overweight was between the 85th and 95th percentile, and obese was greater than or equal to the 95th percentile. Primary disease was defined as the underlying disease (divided as surgical and nonsurgical diseases) prior to diagnosis of COVID-19 of the samples. We excluded mild COVID-19 without underlying disease. Anemia is a condition in which the number of red blood cells or the haemoglobin concentration within them is lower than the lowest reference value for age; 0–30 days < 15.0 g/L, 1–23 months < 10.5 g/L, 2–9 years < 11.5 g/L, 10–17 years male < 12.5 g/L, and 10–17 years female < 12.0 g/L. Thrombocytopenia is defined as a platelet count of <150,000/microL. Leukocytosis is defined as a white blood cell (WBC) count greater than the highest reference value for age. Leukopenia is defined as a WBC count lower than the lowest reference value for age. The WBC count reference value based on age as follows; 0–30 days 9,100–34,000/microL, 1–23 months 6,000–14,000/microL, 2–9 years 4,000–12,000/microL, and 10–17 years 4,000–10,500/microL. All the blood results were taken on the first day of hospitalization.

Classification and management of COVID-19 patients in children was performed using the national COVID-19 treatment protocol made by Indonesian Pediatric Society 2020 which was revised in 2021 and 2022. A moderate COVID-19 infection is classified as a patient with clinical signs of pneumonia: fever, cough, and tachypnea, which may be accompanied by crackles or wheezing without respiratory distress and hypoxemia. Moderate COVID-19 patients were treated in isolation room and were given non-pharmacology therapy (oxygen via nasal canula on NRM, maintenance fluid administration, and optimal nutrition), and pharmacology therapy i.e antiviral (oseltamivir), antibiotic (ceftriaxone and / or azithromycin), corticosteroid, vitamin C, vitamin D3, and zinc. Severe COVID-19 infection is classified as patient with clinical signs of respiratory distress to failure characterized by nasal flaring, cyanosis, subcostal retractions, desaturation <95%, seizures, decrease of consciousness, profuse vomiting, poor feeding, with or without any respiratory signs. The critical COVID-19 infection is classified as a patient with progresses rapidly to acute respiratory distress syndrome (ARDS) or respiratory failure or develops shock, encephalopathy, myocardial damage or heart failure, coagulopathy, acute renal impairment, and multiple organ dysfunction or other manifestation of sepsis. Severe and critical COVID-19 patients are isolated in the isolation room with negative pressure ventilation, treated with non-pharmacology therapy (maintenance fluid administration and optimal nutrition), and pharmacology therapy i.e antiviral (oseltamivir), antibiotic (ceftriaxone and / or azithromycin), corticosteroid, vitamin C, vitamin D3, and zinc, high pressure oxygen, with non-invasive ventilation: CPAP or high flow nasal cannula (HFNC) for severe COVID-19, and invasive mechanical ventilator for critical COVID-19 [10]. Indonesian Pediatric Society revised antiviral therapy in 2021 (moderate COVID 19 patient was treated with oseltamivir; severe COVID-19 patient was treated with remdesivir) and 2022 (antiviral was revised to favipiravir and ramdesevir) [10–12].

ARDS was established based on Pediatric Acute Lung Injury Consensus Conference (PALICC). Diagnostic criteria for noninvasive mechanical ventilation: Oxygen Index (OI) (PaO2/FiO2 ratio) ≤ 300 or Saturation Index (OSI) (SpO2/FiO2 ratio) ≤ 264. In cases with invasive mechanical ventilation, ARDS is considered as mild if: 4 ≤ OI < 8 or 5 ≤ OSI < 7.5; moderate 8 ≤ OI < 16 or 7.5 ≤ OSI < 12.3; and severe if OI > 16 or OSI >12.3 [13]. Acute Kidney Injury (AKI) is diagnosed based on Kidney Disease Improving Global Outcomes (KDIGO) criteria as: Stage 1: Serum creatinine 1.5–1.9 times the baseline value, or an increase of ≥ 0.3 mg/dL or urine output < 0.5 mL/kgBW/hour within 6–12 hours, Stage 2: Serum creatinine 2.0–2.9 times baseline or urine output < 0.5 mL/kg/hour for ≥ 12 hours, Stage 3: Serum creatinine 3.0 times baseline, or serum creatinine elevation ≥ 4.0 mg/dL, or initiation of renal replacement therapy, or in patients < 18 years, decreased GFR < 35 mL/min per 1.73 m^2^ or urine output < 0.3 mL/kg/hour for ≥ 24 hours, or anuria for 12 hours [14]. Laboratories cut off were as follows: neutrophil-lymphocyte count ratio (NLR) is 3 [15], absolute lymphocyte count (ALC) is 1500 cells/uL [16], PT 13 seconds, and APTT is 42 seconds [17].

### Statistical analysis

Frequency distributions, mean values, standard deviations, medians, and ranges were analyzed using SPSS version 21. To determine normal distribution of numeric data, Kolmogorov-Smirnov was applied, if p <0.05, the data distribution considered as not normal. Data variance was tested using Levene’s test. Bivariate analysis used Chi-Square test, Independent T-test, or Mann Whitney. Chi-Square test was used to analyze categorical variable between independent and dependent variables, also to calculate odds ratio (OR) and Confidence Interval (CI) value. Independent T-test was used to compare numeric variable in 2 unpair groups with normal data distribution. If data was not in normal distribution, we used alternative test, i.e Mann-Whitney-U test. If there was more than one data in bivariate analysis with p<0.05. Multivariate analysis will be performed with logistic multiple regression analysis. To predict probability of COVID-19 patients with non-survive, we establish the cut point of IL-6 through optimal point between sensitivity and specificity, and Receiver Operating Characteristic (ROC) analysis (ROC figure, area under the curve (AUC) value, and CI). Meanwhile, to calculate the mortality probability we used formula as follow as: $=\frac{1}{1+\;e^{-y}}$. Interpretation of hypothesis test was considered as significant if p<0.05.

## Results

**Fig 1:**
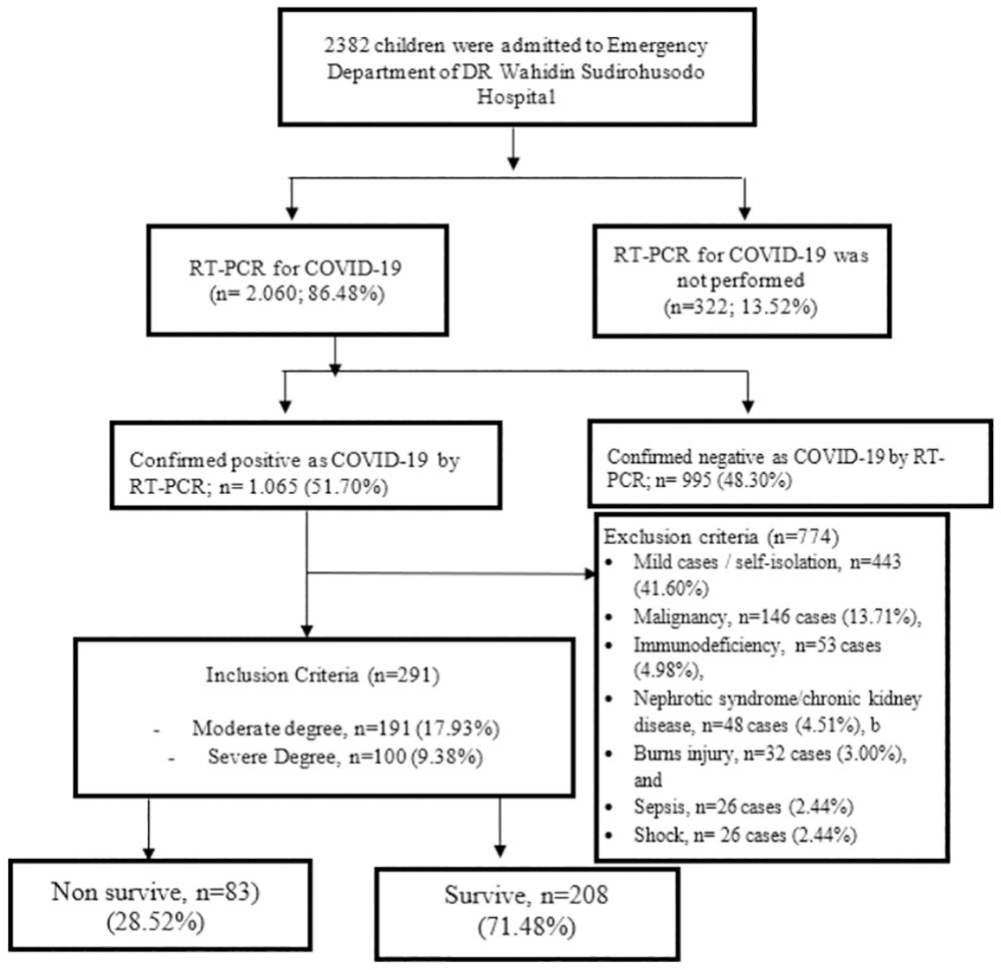
Research flowchart.

To There were 2386 patients being admitted to emergency department, and 2060 patients underwent nasopharyngeal and oropharyngeal swab. From 2060 patient, there were 1065 was confirmed as positive COVID-19. From 1065 cases recruited, only 291 patients met the inclusion criteria and classified as moderate COVID-19 in 191 patients (17.94%) and severe COVID-19 in 100 patients (9.38%). From 291 cases, 83 (28.52%) patients were not survived, and 208 (71.48%) patients survived. Exclusion criteria were in 774 patients, included 443 (41.60%) mild cases who underwent self-isolation at home, malignancy in 146 cases (13.71%), immunodeficiency in 53 cases (4.98%), nephrotic syndrome/chronic kidney disease in 48 cases (4.51%), burns injury in 32 cases (3.00%), sepsis in 26 cases (2.44%), and shock in 26 cases (2.44%) (Fig 1).

The parameter affected mortality are malnutrition, LOS ≥ 10 days, oxygen saturation <95%, leukopenia/leukocytosis, decreased ALC, prolonged PT/aPTT, AKI, ARDS, and increased of IL-6 level (p<0.05). From the parameter affected mortality, the highest percentage was ARDS in 71.11% cases [OR 3.45 (95% CI 1.93–6.16)] and followed by AKI in 61.67% [OR 5.08 (95% CI 2.44–10.73)]. The Mann-Whitney test showed a significant difference between IL-6 levels in the non-survive and the surviving COVID-19 groups (p 0.00) (Table 1).

**Table 1:** The relationship between demographics, clinical and laboratory parameters with mortality in pediatric patients with COVID-19.

Variable	Non-survive n = 83	Survive n = 208	p	OR(95%CI)
**Sex**				
Male **n = 183**	53 (28.97%)	130 (71.03%)	0.82*	1.06 (0.62–1.79)
Female **n = 108**	30 (27.77%)	78 (72.23%)		
**Age**				
< 5 Years old **n = 145**	45 (31.03%)	100 (68.97%)	0.34*	1.27 (0.76–2.13)
≥5 Years old **n = 146**	38 (26.02%)	108 (73.98%)		
**Nutritional status**				
Normal **n = 206**	51 (24.75%)	155 (75.25%)	0.02*	0.54 (0.31–0.93)
Malnutrition **n = 85**	32 (37.64%)	53 (62.36%)		
**Days at symptoms before hospitalization**				
Mean (SD)	4.7 (1.1)	4.0 (1.0)	0.09**	
Median (Min-Max)	5.0 (3–6)	4.0 (2–6)		
**Length of Stay**				
10 days **n = 168**	57 (33.92%)	111 (66.08%)	0.02*	1.91 (1.12–3.28)
< 10 days **n = 123**	26 (21.13%)	97 (78.87%)		
**Primary Disease**				
Surgical **n = 103**	27 (26.21%)	76 (73.79%)	0.51*	0.83 (0.44–1.43)
Non-Surgical **n = 188**	56 (29.87%)	132 (70.13%)		
**Glasgow Coma Scale (GCS)**				
GCS 15 **n = 221**	57 (25.79%)	164 (74.21%)	0.06*	0.58 (0.33–1.04)
GCS ≤14 **n = 70**	26 (37.14%)	44 (62.86%)		
**Oxygen Saturation**				
95% **n = 122**	26 (21.31%)	96 (78.69%)	0.02*	2.60 (1.42–4.74)
< 95% **n = 179**	57 (33.72%)	112 (66.28%)		
**Hemoglobin**				
Anemia **n = 156**	44 (28.20%)	112 (71.80%)	0.89*	0.96 (0.58–1.61)
Normal **n = 135**	39(28.88%)	96 (71.12%)		
**Leucocyte**				
Leukocytosis/leukopenia **n = 172**	58 (33.72%)	114 (66.28%)	0.02*	1.91 (1.12–3.29)
Normal **n = 119**	25 (21.00%)	94 (79.0%)		
**Platelet**				
Thrombocytopenia **n = 61**	16 (26.22%)	45 (73.78%)	0.65*	0.86 (0.45–1.63)
Normal **n = 230**	67 (29.13%)	163 (70.87%)		
**NLR**				
Increased **n = 152**	50 (32.89%)	102 (67.11%)	0.08*	1.57 (0.92–2.64)
Normal **n = 139**	33 (23.74%)	106 (78.26%)		
**ALC**				
Decreased **n = 119**	43(36.13%)	76 (63.87%)	0.02*	1.87 (1.12–3.14)
Normal **n = 172**	40 (23.25%)	132 (76.75%)		
**PT and APTT**				
Prolonged **n = 86**	41 (46.67%)	45 (52.225)	0.00*	3.53 (2.016–6.08)
Normal **n = 205**	42 (20.48%)	163 (79.52%)		
**Acute Kidney Injury**				
Yes **n = 34**	21 (61.67%)	13 (38.33%)	0.00*	5.08 (2.44–10.73)
No **n = 257**	62 (39.49%)	195 (60.15%)		
**ARDS**				
Yes **n = 45**	32 (71.11%)	13 (28.89%)	0.00*	3.45 (1.93–6.16)
No **n = 246**	51 (20.73%)	195 (79.27%)		

*Chi-square.

**Mann-Whitney U test

IL-6 levels increased in all patients with values varying between 23.48–252.58 pq/ml. The highest levels of IL-6 were found in COVID-19 patient with AKI with a median (SD) of 91.16 (77.89–166.88) pq/ml, ARDS (SD) 83.65 (25.15–252.58), oxygen saturation <95% (SD) 81.09 (45.08–252.58), and thrombocytopenia (SD) of 80.73 (51.3–52.58). Mann-Whitney test showed that there was a significant difference between IL-6 levels in the LOS, oxygen saturation, leukopenia/leukocytosis, AKI, and ARDS (p 0.05) (Table 2).

**Table 2:** Interleukin-6 level in pediatric patients with COVID-19.

Variables	Interleukin-6. levels (pg/ml)		p*
	Mean (SD)	Median (minimum—maximum)	
**Sex**			
Boys	77.38 (32.10)	71.27 (23.48–166.88)	0.34
Girls	81.123 (34.01)	75.76 (49.53–252.58)	
**Age**			
<5-year-old	82.54 (34.64)	72.62 (45.08–252.58)	0.27
≥ 5-year-old	75.44 (30.79)	67.97 (23.48–157.29)	
**Nutritional Status**			
Malnutrition	90.93 (44.72)	79.84 (25.15–252.58)	0.06
Normal	73.52 (24.19)	69.38 (23.48–149.44)	
***Length of stay***			
≥10 days	84.08 (35.50)	75.23 (25.15–252.58)	0.03
<10 days	68.44 (23.51)	67.23 (23.48–157.29)	
**Primary Disease**			
Surgical	72.09 (25.39)	67.49 (23.48–129.34)	0.14
Non-Surgical	82.02 (35.76)	78.92 (25.15–252.58)	
**Glasgow Coma Scale (GCS)**			
GCS 15	78.61 (33.95)	71.74 (23.48–252.58)	0.72
GCS ≤ 14	80.25 (27.89)	75.23 (45.08–166.88)	
**Oxygen Saturation**			
<95%	91.80 (38.56)	81.09 (45.08–252.58)	0.01
≥ 95%	72.58 (27.66)	69.60 (23.48–157.29)	
**Hemoglobin**			
Anemia	81.58 (38.81)	69.32 (25.15–252.58)	0.90
Normal	76.83 (27.29)	72.22 (23.48–166.8)	
**Leukocyte**			
Leukocytosis/leukopenia	82.62 (28.02)	71.82 (23.48–166.88)	0.04
Normal	74.75 (37.27)	67.97 (25.15–252.58)	
**Platelet**			
Thrombocytopenia	100.03 (59.28)	80.73 (51.3–252.58)	0.32
Normal	76.04 (26.59)	71.27 (23.48–166.88)	
**NLR**			
Increased	81.50 (31.26)	71.95 (23.48–166.88)	0.34
Normal	77.53 (33.70)	70.80 (25.15–252.58)	
**ALC**			
Decreased	84.57 (31.29)	80.47 (40.71–16.88)	0.17
Normal	76.94 (33.24)	70.05 (23.48–252.58)	
**PT and APTT**			
Prolonged	87.95 (41.96)	78.54 (45.08–252.58)	0.06
Normal	75.91 (28.78)	67.97 (23.48–157.29)	
**Acute Kidney Injury**			
Yes	102.73 (26.53)	91.16 (77.89–166.88)	0.00
No	72.59 (31.44)	67.60 (23.48–252.58)	
**ARDS**			
Yes	94.48 (37.39)	83.65 (25.15–252.58)	0.00
No	68.55 (24.57)	67.49 (23.48–157.29)	

*Mann-Whitney U test

**Fig 2:**
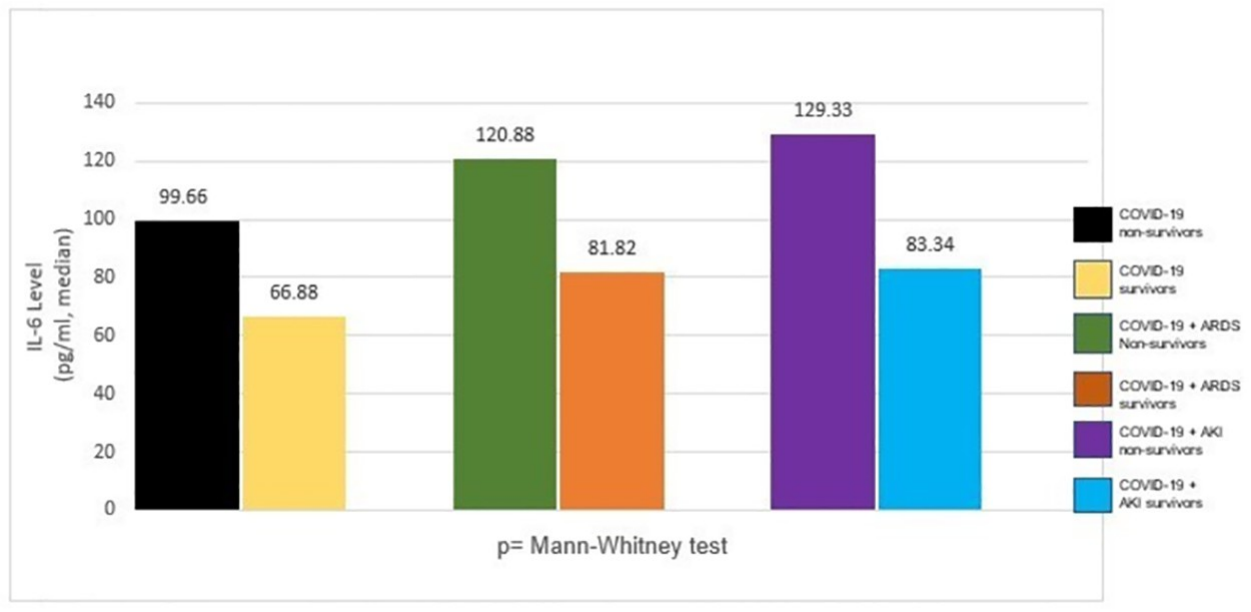
IL-6 levels in COVID-19, ARDS, and AKI non-survivors and survivors.

The median (minimum-maximum) IL-6 levels in COVID-19 non-survived group was 99.66 pg/ml (77.89–252.58) which was higher than the survival group as high as 66.88 pg/ml (23.48–87.39). IL-6 levels in COVID-19 and ARDS non-survives and survives were 120.88 (81.09–252.58) and 81.82 (25.15–88.78), respectively. In addition, IL-6 levels in COVID-19 and AKI non-survives and survives were 129.33 (99.04–166.88) and 83.34 (77.88–99.06), respectively. The Mann-Whitney test showed a significant difference in these three groups (p 0.00) (Fig 2).

**Fig 3:**
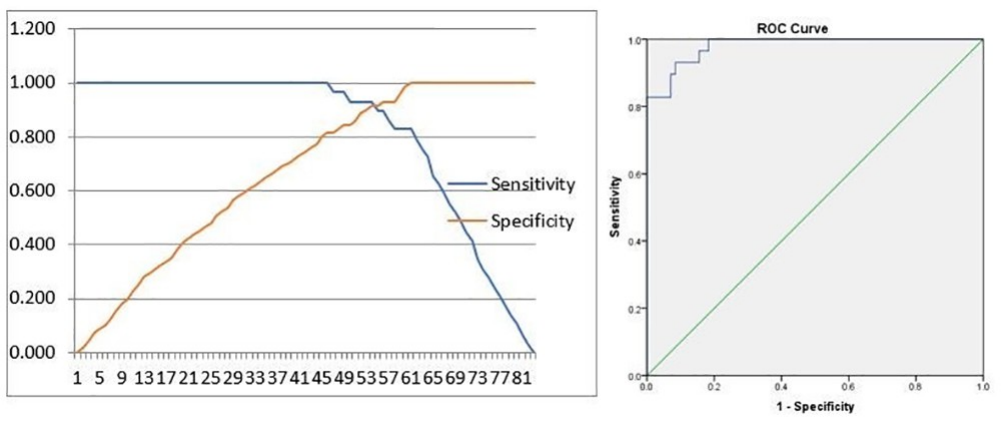
Cut off point and ROC curve of IL-6.

The IL-6 cut-off point of >80.97 pg/ml was the most optimal value as a risk factor for COVID19 mortality in children with sensitivity of 93%, specificity of 90%, positive predictive value of 79% and negative predictive value of 96%. The area under the curve (AUC) 0.981 (95% CI, 0.960–1.000) (Fig 3).

In multivariate analysis with logistic regression analysis, risk factors for COVID-19 mortality in children were IL-6 levels with OR 18.570 (95% CI 5.320–64.803), ARDS OR 10.177, (95% CI 1.310–9.040) and AKI with OR 3.220 (95% CI 1.070–10.362) (Table 3). The increased IL-6 with ARDS, increased of IL-6 with AKI, and increased of IL-6 with ARDS and AKI showed the mortality probability as high as 64.5, 85.4, and 98.3%, respectively.

**Table 3:** Multivariate analysis of risk factor mortality COVID 19 on children.

Variabel	B	SE	Df	Sig	Exp (B)	95% CI for Exp (B)
						Lower-Upper
**ARDS**	4.632	2.221	1	0.037	10.177	1.310–79.040
**AKI**	3.472	1.771	1	0.040	3.220	1.070–10.362
**Leucocyte**	-0.101	1.751	1	0.954	0.904	0.020–27.970
**Nutritional Status**	3.168	2.491	1	0.203	2.376	0.180–31.326
**PT /aPTT**	-0.288	1.890	1	0.879	0.750	0.010–3.052
**Interleukin-6**	9.829	4.162	1	0.018	18.570	5.320–64.803
**ALC**	-5.687	3.609	1	0.115	0.003	0.00–4.002
**Oxygen saturation**	0.544	2.504	1	0.828	1.723	0.010–23.167
**Length of stay**	0.921	1.913	1	0.630	2.512	0.050–10.673
**Constant**	-21.951	9.748	1	0.024	0.000	

## Discussion

For Rapid SARS-CoV-2 replication and viremia lead to overactivation of T cells (immune dysfunction), systemic inflammatory responses and dysfunction of the renin angiotensin system (RAS) which triggers excessive release of proinflammatory cytokines and chemokines (Cytokine Storm) especially IL- 6 which causes damage to various organs resulting in ARDS, acute renal impairment, coagulation disorders and vascular endothelial dysfunction [18, 19].

This study reported the mortality rate of COVID-19 in children was 28.52% and all of them reported with increasing IL-6 levels. All cases also reported with pneumonia, and in 45 (20.54%) cases developed to ARDS and in 44 patients (15.12%) suffer from AKI. Study and case report from Maggio, et al support the strong correlation between interleukin-6 levels and severe clinical manifestations such as COVID-19 pneumonia, and this marker predicts a more severe clinical outcome in children. Testing serum levels of interleukin-6 in children with COVID-19 could be useful to better understand the outcome of lung damage [20].

This study found an association between IL-6 and mortality with cut-off point of 80.97 pg/ml as a predictor. A previous study found that IL-6 serum level was significantly increased in COVID-19 children. Based on that study, IL-6 serum level ≥ 24.3 pg/ml were associated with a greater likelihood of progression to critical illness status. Besides, it showed the sensitivity of interleukin-6 was 87.9% and specificity was 63.5%, at AUC of 0.640 with cut-off of 7.41 [21]. The difference results from our study could be caused by difference in sample size and the severity degree of disease. Our sample’s size is larger and we included only moderate and severe disease, while the previous one included small size of sample and the severity of disease from asymptomatic, mild, moderate, and severe. Increased levels of IL-6 at the initial of treatment describe an inflammatory condition in the lungs that causes damage to the vascular endothelium. diffuse alveolar epithelium, alveolar edema, microthrombi and the development of infection that will lead to hypoxemia, respiratory failure and death [5, 6]. IL-6 is particularly important because of its pleiotropic effects, regulates the inflammatory response, major trigger for cytokine storms and contributes to host defense through stimulation of the acute phase response [4, 21, 22].

In this study, AKI was found in 44 patients (15.12%) with non-survive patients in 21 patients (61.67%) and has the highest mean level of IL-6 which is 102.73 (26.53) pq/ml. Study from Kari et al [23] showed AKI occurred in one-fifth of children with SARS-CoV-2 infection requiring hospital admission, with one-third of those requiring PICU. AKI was significantly associated with more frequent ICU admission (32 % vs. 2.8 %, *p* < 0.001) and mortality (42 % vs. 0 %, *p* < 0.001), but not with prolonged hospitalization (58 % vs. 40 %, *p* = 0.163).

Our study is in accordance with another work from Chopra, et al, found that AKI is an important modifiable risk factor for mortality in children with COVID19 in a resource-limited setting. Mortality (25.71%) was higher in the AKI group (OR 95% CI, 1.14–8.35, p < 0.023) with shock (OR 45.92; 95% CI, 3.44–612.0, p value <0.004) and ventilation (OR 46.24; 95% CI, 1.6–1333.0 p value< 0.02) as significant risk factors for mortality [24]. But the difference in mortality rate in AKI from our study to Chopra’s is probably caused by different in sample size, methodology of study, exclusion criteria, and antiviral treatment. The cause of AKI in children is still unknown. it is thought to be a direct effect of SARS-CoV-2 infection, cytokine storm (IL-6). hypoxemia and dehydration or shock [25]. In this study several patients reported with AKI. However, it is difficult to determine the most definitive cause of AKI; it can be contributed from SARS-CoV-2 infection, IL-6 cytokine storm, or drug used in patients during admission and before admission. Kidney damage in COVID-19 is secondary to podocyte dysfunction causes glomerular diseases such as focal segmental glomerulosclerosis (FSGS), and acute proximal tubular injury or tubular necrosis, as well as RAAS activation that triggers glomerular dysfunction, fibrosis, vasoconstriction, and inflammation. Activation of coagulation resulted renal vascular injury i.e ischemic glomeruli and fibrinoid necrosis [25].

In this study, ARDS became a risk factor for mortality in COVID-19 patients in 45 (20.54%) and non-survive in 32 cases (71.11%). Meta-analysis from Shi et al [26] reviewed that ARDS (OR = 29.54, 95% CI 12.69–68.78) and AKI (OR = 55.02, 95% CI 6.26–483.35) increased the odds to be admitted to intensive care; shortness of breath (OR = 16.96, 95% CI 7.66–37.51) increased the need of respiratory support; and neurological diseases (OR = 5.16, 95% CI 2.30–11.60). This study interprets that ARDS and AKI are associated with unfavorable prognosis in children and adolescents with COVID-19.

One of the strengths of this study is a prospective cohort method that was conducted since the COVID-19 Pandemic period at Wahidin Sudhirohusodo Hospital. Makassar as the main referral hospital in eastern Indonesia, so the subjects of the study came from mostly eastern region of Indonesia and reflected factors that affect the outcome of COVID-19 in children. Treatment or therapeutic intervention uses the same protocol from the Indonesian Pediatrician Society.

The limitations of this study including the examination of interleukin-6 level which was only carried out at the beginning of treatment, thus the changes in interleukin-6 levels in patients with clinical deterioration is not known. IL-6 could not to be test in serial examination because regulation from hospital to limit the contact with COVID-19 patients. Other limitation is the onset of the primary disease course in each patient is vary. Treatment of COVID-19 in red zone areas with very strict regulations making it difficult for the authors to observe the patients regularly. The revision of COVID-19 management protocol makes modification in antiviral regiments, so they are treated with different antiviral therapy. Most of patients have been given medical drugs and traditional or herbal medicines prior to hospitalization. In addition, this study did not sub-divided the available data into the type of COVID-19 strains.

This study concludes that IL-6, ARDS, and AKI are risk factors for death in children with COVID-19. IL-6 level was the highest mortality risk factor. We recommend a serial examination of IL-6 level at the admission and during hospitalization in COVID-19 children as well as a larger and multicenter study on risk factors for COVID-19 mortality in children. The IL-6 inhibitors administration might improve the outcome of patient with high IL-6 levels. In addition, we suggest to perform other research to explore other inflammatory and pro-inflammatory cytokines in COVID-19, study in vaccinated COVID-19 patients and second or third onset of COVID-19 is also needed. It is also needed to perform study of IL-6 levels in mild degree compared with moderate and severe degree of COVID 19. In case of AKI, we recommend in vivo study to establish the role of high IL-6 levels to kidney dysfunction.

## Supporting information

S1 Dataset (PDF)
