# Supplementary material for: IL-6 serum level, ARDS, and AKI as risk factors for the COVID-19 infection’s mortality in children
Source: PLoS One. 2023 Oct 27;18(10):e0293639. doi: 10.1371/journal.pone.0293639 (PMC10610523; doi:10.1371/journal.pone.0293639)
Supplement: S1 Dataset — (PDF) [file pone.0293639.s001.pdf]

Appendix

Gender

Case Processing Summary

		Cases					
		Valid		Missing		Total	
	Gender	N	Percent	N	Percent	N	Percent
interleukin6	Boys	183	100.0%	0	0.0%	183	100.0%
	Girl	108	100.0%	0	0.0%	108	100.0%

Descriptives

	Gender			Statistic	Std. Error
interleukin6	Boys	Mean		77.3888	4.14431
		95% Confidence Interval for Mean	Lower Bound	69.0961	
			Upper Bound	85.6816	
		5% Trimmed Mean		75.8508	
		Median		71.2726	
		Variance		1030.520	
		Std. Deviation		32.10172	
		Minimum		23.48	
		Maximum		166.88	
		Range		143.40	
		Interquartile Range		25.61	
		Skewness		1.051	.309
		Kurtosis		.877	.608
	Girl	Mean		81.2318	5.37873
		95% Confidence Interval for Mean	Lower Bound	70.3523	
			Upper Bound	92.1113	
		5% Trimmed Mean		76.8456	
		Median		75.7650	
		Variance		1157.231	
		Std. Deviation		34.01810	
		Minimum		49.53	
		Maximum		252.58	
		Range		203.06	
		Interquartile Range		28.45	
		Skewness		3.462	.374
		Kurtosis		16.370	.733

Age

Case Processing Summary

		Cases					
		Valid		Missing		Total	
	Age	N	Percent	N	Percent	N	Percent
interleukin6	< 5 year	145	100.0%	0	0.0%	145	100.0%
	> 5 year	146	100.0%	0	0.0%	146	100.0%

Descriptives

	Age			Statistic	Std. Error
interleukin6	< 5 year	Mean		82.5474	4.94964
		95% Confidence Interval for Mean	Lower Bound	72.5955	
			Upper Bound	92.4993	
		5% Trimmed Mean		78.1450	
		Median		72.6255	
		Variance		1200.447	
		Std. Deviation		34.64747	
		Minimum		45.08	
		Maximum		252.58	
		Range		207.50	
		Interquartile Range		25.53	
		Skewness		2.952	.340
		Kurtosis		11.773	.668
	> 5 year	Mean		75.4467	4.31167
		95% Confidence Interval for Mean	Lower Bound	66.7865	
			Upper Bound	84.1069	
		5% Trimmed Mean		74.0265	
		Median		67.9752	
		Variance		948.114	
		Std. Deviation		30.79145	
		Minimum		23.48	
		Maximum		157.29	
		Range		133.81	
		Interquartile Range		32.87	
		Skewness		.913	.333
		Kurtosis		.764	.656

Nutritional Status

Case Processing Summary

	NutritionalStatus	Cases Valid N	Valid Percent	Missing N	Missing Percent	Total N	Total Percent
interleukin6	malnutrisi	85	100.0%	0	0.0%	85	100.0%
	normal	206	100.0%	0	0.0%	206	100.0%

Descriptives

	NutritionalStatus			Statistic	Std. Error
interleukin6	malnutrisi	Mean		73.5296	2.91218
		95% Confidence Interval for Mean	Lower Bound	67.7184	
			Upper Bound	79.3407	
		5% Trimmed Mean		72.4030	
		Median		69.3885	
		Variance		585.175	
		Std. Deviation		24.19039	
		Minimum		23.48	
		Maximum		149.44	
		Range		125.96	
		Interquartile Range		23.22	
		Skewness		.946	.289
		Kurtosis		1.454	.570
	normal	Mean		90.9375	8.03308
		95% Confidence Interval for Mean	Lower Bound	74.5318	
			Upper Bound	107.3433	
		5% Trimmed Mean		86.8212	
		Median		79.8487	
		Variance		2000.443	
		Std. Deviation		44.72631	
		Minimum		25.15	
		Maximum		252.58	
		Range		227.43	
		Interquartile Range		35.36	
		Skewness		1.839	.421
		Kurtosis		4.644	.821

Case Processing Summary

		Cases					
		Valid		Missing		Total	
	LoS	N	Percent	N	Percent	N	Percent
interleukin6	< 10 day	123	100.0%	0	0.0%	123	100.0%
	> 10 day	168	100.0%	0	0.0%	168	100.0%

Descriptives

	LoS			Statistic	Std. Error
interleukin6	< 10 dayi	Mean		84.0878	4.33731
		95% Confidence Interval for Mean	Lower Bound	75.4281	
			Upper Bound	92.7475	
		5% Trimmed Mean		81.0523	
		Median		75.2339	
		Variance		1260.422	
		Std. Deviation		35.50242	
		Minimum		25.15	
		Maximum		252.58	
		Range		227.43	
		Interquartile Range		29.60	
		Skewness		2.031	.293
		Kurtosis		6.760	.578
	> 10 day	Mean		68.4460	4.09288
		95% Confidence Interval for Mean	Lower Bound	60.1091	
			Upper Bound	76.7830	
		5% Trimmed Mean		67.2330	
		Median		67.4533	
		Variance		552.805	
		Std. Deviation		23.51181	
		Minimum		23.48	
		Maximum		157.29	
		Range		133.81	
		Interquartile Range		26.79	
		Skewness		1.385	.409
		Kurtosis		5.661	.798

Case Processing Summary

	Disease	Cases					
		Valid		Missing		Total	
		N	Percent	N	Percent	N	Percent
interleukin6	surgical	103	100.0%	0	0.0%	103	100.0%
	non surgical	188	100.0%	0	0.0%	188	100.0%

Descriptives

	Disease			Statistic	Std. Error
interleukin6	surgical	Mean		72.9040	4.35487
		95% Confidence Interval for Mean	Lower Bound	64.0439	
			Upper Bound	81.7640	
		5% Trimmed Mean		72.1588	
		Median		67.4944	
		Variance		644.806	
		Std. Deviation		25.39303	
		Minimum		23.48	
		Maximum		129.34	
		Range		105.86	
		Interquartile Range		31.53	
		Skewness		.772	.403
		Kurtosis		.304	.788
	non surgical	Mean		82.0283	4.40228
		95% Confidence Interval for Mean	Lower Bound	73.2363	
			Upper Bound	90.8203	
		5% Trimmed Mean		78.9214	
		Median		72.2284	
		Variance		1279.082	
		Std. Deviation		35.76426	
		Minimum		25.15	
		Maximum		252.58	
		Range		227.43	
		Interquartile Range		25.01	
		Skewness		2.181	.295
		Kurtosis		7.419	.582

Case Processing Summary

	GCS	Cases					
		Valid		Missing		Total	
		N	Percent	N	Percent	N	Percent
interleukin6	GCS 15	221	100.0%	0	0.0%	221	100.0%
	GCS < 15	70	100.0%	0	0.0%	70	100.0%

Descriptives

	GCS			Statistic	Std. Error
interleukin6	GCS 15	Mean		78.6139	3.77285
		95% Confidence Interval for Mean	Lower Bound	71.1057	
			Upper Bound	86.1221	
		5% Trimmed Mean		75.9723	
		Median		71.7429	
		Variance		1152.985	
		Std. Deviation		33.95563	
		Minimum		23.48	
		Maximum		252.58	
		Range		229.10	
		Interquartile Range		28.92	
		Skewness		2.093	.267
		Kurtosis		7.859	.529
	GCS ≤ 14	Mean		80.2567	6.39966
		95% Confidence Interval for Mean	Lower Bound	66.8116	
			Upper Bound	93.7019	
		5% Trimmed Mean		77.3988	
		Median		75.2339	
		Variance		778.156	
		Std. Deviation		27.89545	
		Minimum		45.08	
		Maximum		166.88	
		Range		121.80	
		Interquartile Range		12.96	
		Skewness		2.007	.524
		Kurtosis		4.716	1.014

Case Processing Summary

	Saturation	Cases Valid N	Valid Percent	Missing N	Missing Percent	Total N	Total Percent
interleukin6	< 95	179	100.0%	0	0.0%	179	100.0%
	> 95	122	100.0%	0	0.0%	122	100.0%

Descriptives

	Saturation			Statistic	Std. Error
interleukin6	< 95	Mean		91.8090	6.71258
		95% Confidence Interval for Mean	Lower Bound	78.1359	
			Upper Bound	105.4821	
		5% Trimmed Mean		86.9878	
		Median		81.0961	
		Variance		1486.939	
		Std. Deviation		38.56084	
		Minimum		45.08	
		Maximum		252.58	
		Range		207.50	
		Interquartile Range		27.26	
		Skewness		2.662	.409
		Kurtosis		9.030	.798
	> 95	Mean		72.5807	3.38039
		95% Confidence Interval for Mean	Lower Bound	65.8315	
			Upper Bound	79.3299	
		5% Trimmed Mean		71.1656	
		Median		67.6011	
		Variance		765.612	
		Std. Deviation		27.66970	
		Minimum		23.48	
		Maximum		157.29	
		Range		133.81	
		Interquartile Range		28.64	
		Skewness		1.139	.293
		Kurtosis		1.454	.578

Case Processing Summary

	Hb	Cases					
		Valid		Missing		Total	
		N	Percent	N	Percent	N	Percent
interleukin6	anemia	156	100.0%	0	0.0%	156	100.0%
	normal	135	100.0%	0	0.0%	135	100.0%

Descriptives

	Hb			Statistic	Std. Error
interleukin6	anemia	Mean		81.5905	5.85085
		95% Confidence Interval for Mean	Lower Bound	69.7911	
			Upper Bound	93.3898	
		5% Trimmed Mean		77.7535	
		Median		69.3272	
		Variance		1506.228	
		Std. Deviation		38.81015	
		Minimum		25.15	
		Maximum		252.58	
		Range		227.43	
		Interquartile Range		28.63	
		Skewness		2.338	.357
		Kurtosis		7.850	.702
	normal	Mean		76.8326	3.64681
		95% Confidence Interval for Mean	Lower Bound	69.5242	
			Upper Bound	84.1409	
		5% Trimmed Mean		75.3003	
		Median		72.2284	
		Variance		744.755	
		Std. Deviation		27.29021	
		Minimum		23.48	
		Maximum		166.88	
		Range		143.40	
		Interquartile Range		25.45	
		Skewness		1.107	.319
		Kurtosis		2.331	.628

Case Processing Summary

	Leukocyte	Cases					
		Valid		Missing		Total	
		N	Percent	N	Percent	N	Percent
interleukin6	leukositosis dan leukopenia	172	100.0%	0	0.0%	172	100.0%
	normal	119	100.0%	0	0.0%	119	100.0%

Descriptives

	Leukocyte			Statistic	Std. Error
interleukin6	leukositosis dan leukopenia	Mean		82.6275	3.84908
		95% Confidence Interval for Mean	Lower Bound	74.9037	
			Upper Bound	90.3512	
		5% Trimmed Mean		80.9077	
		Median		71.8268	
		Variance		785.218	
		Std. Deviation		28.02175	
		Minimum		23.48	
		Maximum		166.88	
		Range		143.40	
		Interquartile Range		25.64	
		Skewness		1.129	.327
		Kurtosis		1.260	.644
	normal	Mean		74.7520	5.43700
		95% Confidence Interval for Mean	Lower Bound	63.8079	
			Upper Bound	85.6962	
		5% Trimmed Mean		70.7452	
		Median		67.9752	
		Variance		1389.365	
		Std. Deviation		37.27419	
		Minimum		25.15	
		Maximum		252.58	
		Range		227.43	
		Interquartile Range		27.68	
		Skewness		2.704	.347
		Kurtosis		10.794	.681

Case Processing Summary

	platelet	Cases					
		Valid		Missing		Total	
		N	Percent	N	Percent	N	Percent
interleukin6	trombositopenia	61	100.0%	0	0.0%	61	100.0%
	normal	230	100.0%	0	0.0%	230	100.0%

Descriptives

	platelet			Statistic	Std. Error
interleukin6	trombositopenia	Mean		100.0353	17.11486
		95% Confidence Interval for Mean	Lower Bound	62.3658	
			Upper Bound	137.7049	
		5% Trimmed Mean		94.2683	
		Median		80.7320	
		Variance		3515.022	
		Std. Deviation		59.28762	
		Minimum		51.30	
		Maximum		252.58	
		Range		201.29	
		Interquartile Range		74.13	
		Skewness		1.729	.637
		Kurtosis		3.298	1.232
	normal	Mean		76.0475	2.83529
		95% Confidence Interval for Mean	Lower Bound	70.4120	
			Upper Bound	81.6829	
		5% Trimmed Mean		74.6485	
		Median		71.2726	
		Variance		707.420	
		Std. Deviation		26.59736	
		Minimum		23.48	
		Maximum		166.88	
		Range		143.40	
		Interquartile Range		24.41	
		Skewness		1.084	.257
		Kurtosis		1.837	.508

Case Processing Summary

		Cases					
		Valid		Missing		Total	
	NLR	N	Percent	N	Percent	N	Percent
interleukin6	Increased	152	100.0%	0	0.0%	152	100.0%
	Normal	139	100.0%	0	0.0%	139	100.0%

Descriptives

	NLR			Statistic	Std. Error
interleukin6	Increased	Mean		81.5052	5.28448
		95% Confidence Interval for Mean	Lower Bound	70.7659	
			Upper Bound	92.2446	
		5% Trimmed Mean		79.9910	
		Median		71.9522	
		Variance		977.401	
		Std. Deviation		31.26341	
		Minimum		23.48	
		Maximum		166.88	
		Range		143.40	
		Interquartile Range		26.85	
		Skewness		1.118	.398
		Kurtosis		1.223	.778
	Normal	Mean		77.5372	4.18035
		95% Confidence Interval for Mean	Lower Bound	69.1860	
			Upper Bound	85.8884	
		5% Trimmed Mean		74.5808	
		Median		70.8023	
		Variance		1135.895	
		Std. Deviation		33.70305	
		Minimum		25.15	
		Maximum		252.58	
		Range		227.43	
		Interquartile Range		28.09	
		Skewness		2.532	.297
		Kurtosis		10.696	.586

Case Processing Summary

	ALC	Cases					
		Valid		Missing		Total	
		N	Percent	N	Percent	N	Percent
interleukin6	Decreased	119	100.0%	0	0.0%	119	100.0%
	Normal	172	100.0%	0	0.0%	172	100.0%

Descriptives

	ALC			Statistic	Std. Error
interleukin6	Decreased	Mean		84.5727	6.13743
		95% Confidence Interval for Mean	Lower Bound	71.9324	
			Upper Bound	97.2129	
		5% Trimmed Mean		82.5807	
		Median		80.4724	
		Variance		979.370	
		Std. Deviation		31.29488	
		Minimum		40.71	
		Maximum		166.88	
		Range		126.17	
		Interquartile Range		26.81	
		Skewness		1.136	.456
		Kurtosis		.899	.887
	Normal	Mean		76.9421	3.86467
		95% Confidence Interval for Mean	Lower Bound	69.2398	
			Upper Bound	84.6444	
		5% Trimmed Mean		74.1639	
		Median		70.0519	
		Variance		1105.242	
		Std. Deviation		33.24517	
		Minimum		23.48	
		Maximum		252.58	
		Range		229.10	
		Interquartile Range		24.11	
		Skewness		2.422	.279
		Kurtosis		10.117	.552

Case Processing Summary

	PT/APTT	Cases					
		Valid		Missing		Total	
		N	Percent	N	Percent	N	Percent
interleukin6	Prolonged	86	100.0%	0	0.0%	86	100.0%
	normal	205	100.0%	0	0.0%	205	100.0%

Descriptives

	PT/APTT			Statistic	Std. Error
interleukin6	Prolonged	Mean		87.9557	3.32338
		95% Confidence Interval for Mean	Lower Bound	69.2937	
			Upper Bound	82.5376	
		5% Trimmed Mean		74.6073	
		Median		78.5452	
		Variance		828.364	
		Std. Deviation		41.96131	
		Minimum		45.08	
		Maximum		252.58	
		Range		133.81	
		Interquartile Range		25.77	
		Skewness		.985	.277
		Kurtosis		.816	.548
	normal	Mean		75.9171	8.39340
		95% Confidence Interval for Mean	Lower Bound	70.6340	
			Upper Bound	105.2803	
		5% Trimmed Mean		82.0767	
		Median		67.9729	
		Variance		1761.231	
		Std. Deviation		28.78702	
		Minimum		23.48	
		Maximum		157.29	
		Range		207.50	
		Interquartile Range		19.46	
		Skewness		2.919	.464
		Kurtosis		10.095	.902

Case Processing Summary

	AKI	Cases: Valid N	Valid Percent	Missing N	Missing Percent	Total N	Total Percent
interleukin6	AKI	34	100.0%	0	0.0%	34	100.0%
	Non AKI	257	100.0%	0	0.0%	257	100.0%

Descriptives

	AKI			Statistic	Std. Error
interleukin6	AKI	Mean		102.7342	5.79007
		95% Confidence Interval for Mean	Lower Bound	90.6563	
			Upper Bound	114.8121	
		5% Trimmed Mean		100.5945	
		Median		91.1685	
		Variance		704.023	
		Std. Deviation		26.53344	
		Minimum		77.89	
		Maximum		166.88	
		Range		88.99	
		Interquartile Range		40.14	
		Skewness		1.222	.501
		Kurtosis		.353	.972
	Non AKI	Mean		72.5973	3.53815
		95% Confidence Interval for Mean	Lower Bound	65.5534	
			Upper Bound	79.6412	
		5% Trimmed Mean		69.7168	
		Median		67.6011	
		Variance		988.963	
		Std. Deviation		31.44778	
		Minimum		23.48	
		Maximum		252.58	
		Range		229.10	
		Interquartile Range		24.32	
		Skewness		2.896	.271
		Kurtosis		13.526	.535

Case Processing Summary

	ARDS	Cases					
		Valid		Missing		Total	
		N	Percent	N	Percent	N	Percent
interleukin6	ARDS	45	100.0%	0	0.0%	45	100.0%
	non ARDS	246	100.0%	0	0.0%	246	100.0%

Descriptives

	ARDS			Statistic	Std. Error
interleukin6	ARDS	Mean		94.4821	5.91189
		95% Confidence Interval for Mean	Lower Bound	82.5242	
			Upper Bound	106.4400	
		5% Trimmed Mean		90.9192	
		Median		83.6572	
		Variance		1398.019	
		Std. Deviation		37.39009	
		Minimum		25.15	
		Maximum		252.58	
		Range		227.43	
		Interquartile Range		38.54	
		Skewness		2.118	.374
		Kurtosis		7.432	.733
	non ARDS	Mean		68.5553	3.17261
		95% Confidence Interval for Mean	Lower Bound	62.2069	
			Upper Bound	74.9037	
		5% Trimmed Mean		66.4222	
		Median		67.4944	
		Variance		603.927	
		Std. Deviation		24.57493	
		Minimum		23.48	
		Maximum		157.29	
		Range		133.81	
		Interquartile Range		24.74	
		Skewness		1.703	.309
		Kurtosis		4.300	.608

Area Under the Curve

Test Result Variable(s): interleukin6

Area	Std. Error[a]	Asymptotic Sig.[b]	Asymptotic 95% Confidence Interval	
			Lower Bound	Upper Bound
.981	.011	.000	.960	1.000

a. Under the nonparametric assumption

b. Null hypothesis: true area = 0.5

Coordinates of the Curve

Test Result Variable(s): interleukin6

Positive if Greater Than or Equal To[a]	Sensitivity	1 - Specificity
22.4789	1.000	1.000
24.3147	1.000	.986
32.9294	1.000	.958
42.5995	1.000	.930
44.7849	1.000	.915
47.3023	1.000	.901
50.4106	1.000	.873
51.3491	1.000	.845
51.6482	1.000	.817
52.0311	1.000	.803
52.8093	1.000	.775
53.8385	1.000	.746
55.0984	1.000	.718
56.9723	1.000	.704
59.1592	1.000	.690
60.4328	1.000	.676
61.0531	1.000	.662
61.7209	1.000	.648
61.8889	1.000	.620
62.1573	1.000	.592
63.3524	1.000	.577
64.5885	1.000	.563

65.3073	1.000	.549
65.9668	1.000	.535
66.5361	1.000	.521
67.1700	1.000	.493
67.4944	1.000	.479
67.5683	1.000	.465
67.6502	1.000	.437
67.8049	1.000	.423
67.9428	1.000	.408
67.9913	1.000	.394
68.6367	1.000	.380
69.3272	1.000	.366
69.9057	1.000	.352
70.5690	1.000	.338
70.7587	1.000	.324
71.2726	1.000	.310
71.7849	1.000	.296
71.8895	1.000	.282
72.0008	1.000	.268
72.2284	1.000	.254
72.5164	1.000	.239
73.9297	1.000	.225
75.7650	1.000	.197
77.0922	1.000	.183
77.8937	.966	.183
78.2209	.966	.169
78.7159	.966	.155
79.2174	.931	.155
79.6974	.931	.141
80.3526	.931	.113
80.9763	.931	.099
81.5423	.931	.085
82.0391	.897	.085
82.2179	.897	.070
82.9556	.862	.070
83.6572	.828	.070
85.4253	.828	.042
87.2476	.828	.014

88.0796	.828	.000
88.9198	.793	.000
89.9582	.759	.000
91.0053	.724	.000
91.3426	.655	.000
94.2664	.621	.000
98.0322	.586	.000
99.3575	.552	.000
99.6672	.517	.000
107.0470	.483	.000
118.0588	.448	.000
122.3916	.414	.000
125.3748	.345	.000
128.4987	.310	.000
129.3845	.276	.000
131.0066	.241	.000
132.8107	.207	.000
139.9478	.172	.000
148.1499	.138	.000
153.3658	.103	.000
162.0832	.069	.000
209.7291	.034	.000
253.5816	.000	.000

a. The smallest cutoff value is the minimum observed test value minus 1, and the largest cutoff value is the maximum observed test value plus 1. All the other cutoff values are the averages of two consecutive ordered observed test values.

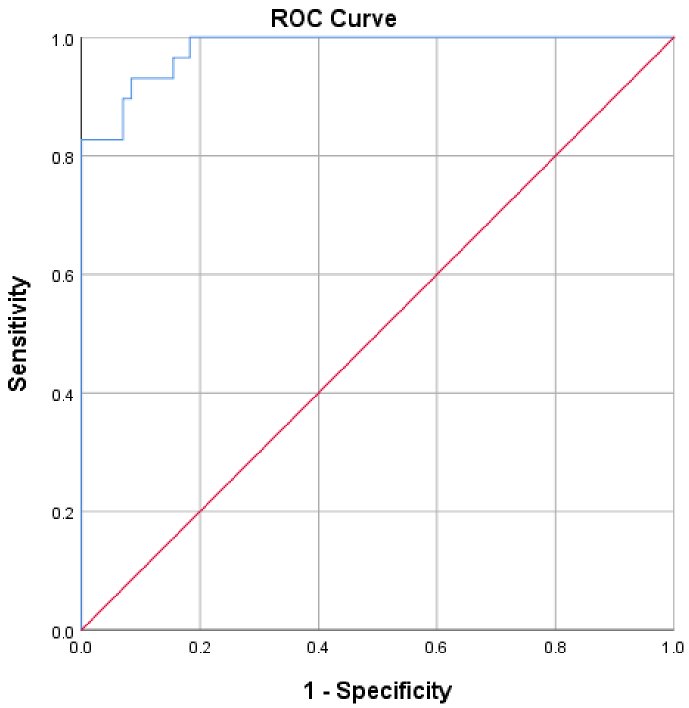
ROC Curve
Sensitivity
1 - Specificity
0.0
0.2
0.4
0.6
0.8
1.0
